# Comparing Intrinsic Catalytic Activity and Practical Performance of Ni- and Pt-Based Alkaline Anion Exchange Membrane Water Electrolyzer Cathodes

**DOI:** 10.1021/acsenergylett.5c00439

**Published:** 2025-03-18

**Authors:** Advay Shirwalkar, Manjodh Kaur, Sichen Zhong, Max Pupucevski, Keda Hu, Yushan Yan, Judith Lattimer, James McKone

**Affiliations:** † Department of Chemical and Petroleum Engineering, University of Pittsburgh, Pittsburgh, Pennsylvania 15261, United States; ‡ Giner Laboratories, Newton, Massachusetts 02466, United States; ¶ Versogen, Inc., Newark, Delaware 19711, United States; § Department of Chemistry, University of Pittsburgh, Pittsburgh, Pennsylvania 15261, United States

## Abstract

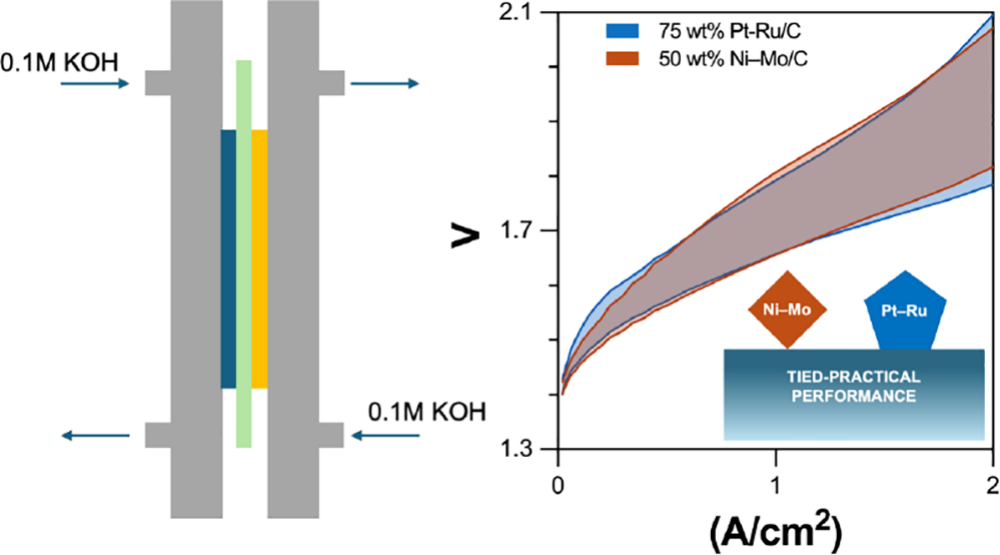

The stringent cost and performance requirements of renewable hydrogen production systems dictate that electrolyzers benefit from the use of nonprecious catalysts only if they deliver the same level of activity and durability as their precious metal counterparts. Here we report on recent work to understand interrelationships between the intrinsic activity of Ni- and Pt-based electrolyzer cathode catalysts and their performance in zero-gap alkaline water electrolyzer assemblies. Our results suggest that nanoparticulate Ni–Mo exhibits HER activity that is roughly 10-fold lower than Pt–Ru on the basis of turnover frequency under low (≤100 mV) polarization conditions. We further found that the HER activity of Ni–Mo/C cathodes is inhibited by aryl piperidinium anion-exchange ionomers bearing bicarbonate counter-anions. After addressing this poisoning effect, we produced electrolyzer assemblies based on Ni–Mo/C cathodes that delivered indistinguishable current density vs cell potential relationships compared to otherwise identical assemblies with Pt–Ru cathodes. This result indicates that the contribution of the cathode to the total cell polarization is small, even for the less active Ni–Mo/C catalyst, and further implies that Pt-based cathodes can indeed be replaced by nonprecious alternatives with no loss in performance.

Anion exchange membrane (AEM) water electrolyzers are under increasingly active development for decarbonized hydrogen production.^1^ The proposed advantages can be summarized as the ability to deliver the performance characteristics (efficiency and H_2_ production rate) of proton-exchange membrane (PEM) electrolyzer systems at a much lower capital cost. The anticipated cost advantages stem primarily from balance-of-plant components and the substitution of Ir-based anode catalysts with nonprecious alternatives based on first-row transition metals like Ni, Fe, and Co.^2^ Notably, replacing industry-standard Pt with nonprecious alternatives like Ni in electrolyzer cathodes is less impactful than replacing replacement of Ir anodes. Consider, for example, that the predicted global annual demand for electrolytic hydrogen is expected to reach 300 million metric tons by 2050.^3^ This demand could be met by using approximately 150 t of platinum, which is comparable to one year of global primary production^4^–a large but not astronomical quantity. (This estimate assumes all electrolytic hydrogen demand is met by PEM electrolyzer systems operating with Pt loading of 0.1 mg/cm^2^ while operating at 2 A cm^–2^ with a capacity factor of 0.33.) Nonetheless, the benefits of nonprecious catalysts extend also to supply chain security and environmental sustainability associated with resource recovery. Thus, a transition from Pt to nonprecious catalysts in water electrolyzer cathodes can be considered as preferable but not imperative. This means nonprecious cathodes need to demonstrate performance characteristics that compare favorably with their platinum-group metal (PGM) counterparts in water electrolyzers to justify their use in commercial systems.

Alongside these technological considerations, the development of nonprecious electrolyzer cathodes motivates interesting fundamental research questions. For example, can nonprecious catalysts be designed that deliver comparable intrinsic catalytic activity to precious metals like Pt? Prior work has suggested that simple alloying of nonprecious metals exhibiting widely disparate hydrogen binding energies should give access to catalysts with continuously tunable activity toward the hydrogen evolution reaction (HER).^5,6^ By this logic, using mixtures of miscible transition metals whose H-binding energies bracket the “volcano” peak demarcating strong versus weak binding (e.g., Ni and Cu), it should be feasible to access an alloy phase with HER activity matching that of Pt, whose H-binding energy is understood to be very near the volcano peak.^7^ Nonetheless, to date there exist no nonprecious catalyst whose mass- or site-specific catalytic activity has been shown to match that of pure Pt nanoparticles.^8^ This is true even after accounting for the markedly lower HER activity exhibited by Pt in alkaline conditions, which is understood to stem from additional activation barriers associated with water dissociation.^9−11^

Clearly, the full scope of the physics and chemistry of nonprecious HER catalysts has not yet been elucidated. Our view is that a major part of the challenge in this field stems from ambiguity in the interpretation of catalytic behavior in terms of physical properties (e.g., activation barriers and site-specific turnover frequencies) versus performance properties (e.g., the overpotential required to achieve a benchmark geometric current density). This Letter summarizes our recent efforts disentangle these parameters by making direct comparisons between Ni-based and Pt-based nanoparticulate catalysts for alkaline hydrogen evolution under conditions commonly used to estimate intrinsic catalytic activity (thin-film rotating disk electrode voltammetry) and in functional AEM water electrolyzer (AEMWE) assemblies. Our primary focus is on Ni–Mo catalyst composites, which we and several others have demonstrated as among the most active nonprecious catalyst systems for alkaline hydrogen evolution in lab scale devices.^12−19^ By extrapolating specific activity data to low mass loadings, we estimate that Ni–Mo composites are at least 10-fold less active than commercial Pt–Ru composites toward the alkaline HER. Nontheless, AEMWE cells that used cathodes loaded with 1 mg cm^–2^ (metals basis) of Ni–Mo/C produced nearly identical current vs overpotential performance to those loaded with 0.15–0.3 mg cm^–2^ of Pt–Ru/C. These results strongly suggest that it is indeed possible to replace platinum-group metals with nonprecious alternatives in AEMWE cathodes without a loss in performance.

Figure 1 depicts the results of a series of measurements, made under analytical conditions using thin-film rotating disk electrode (RDE) voltammetry, comparing four carbon-supported HER catalysts: Ni/C, Ni–Mo/C, Pt/C, and Pt–Ru/C. Each of these was obtained commercially except Ni–Mo/C, which was synthesized as reported previously^12^ with the addition of a hydrothermal treatment to preoxidize the carbon support. This modification allowed us to produce well-dispersed Ni–Mo nanoparticles at 50 wt % metal loading. Full details of the synthesis and characterization are included in the Supporting Information. In each case, the catalysts were formulated into inks comprising alcohol solvents and ion-conducting polymer binders and dropcast on glassy carbon working electrodes. Measurements were made over a range of catalyst mass loadings using preparation and testing conditions that were identical except for the identity of the catalyst materials.

**Figure 1:**
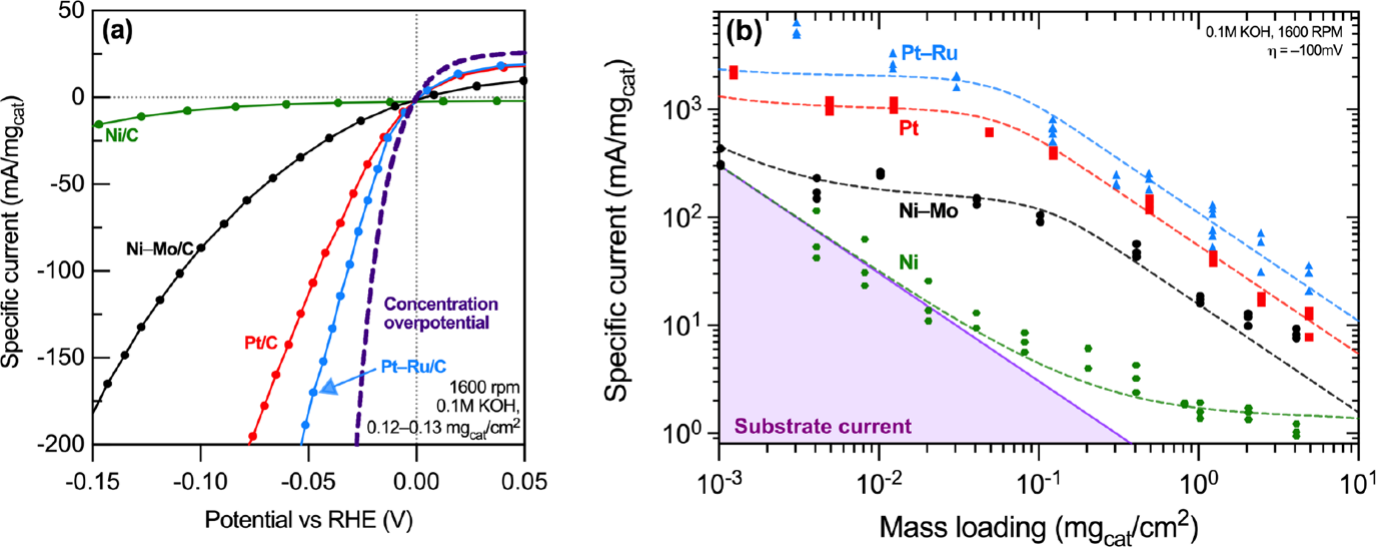
Compiled measurements of HER activity across Ni- and Pt-based catalysts in 0.1 M KOH under thin-film RDE test conditions. (a) Representative polarization curve for the four catalysts of interest at a mass loading of 0.12–0.13 mg_cat_/cm^2^. (b) Cathodic specific current at −100 mV vs RHE versus metal mass loading for the same catalysts and conditions as in panel a. Dashed lines reflect fits to a relationship derived for transport in porous thin films discussed in the Supporting Information.

Figure 1a compiles representative specific current vs overpotential data for all four catalysts loaded at 0.12–0.13 mg_cat_ cm^–2^ (total metals basis) and measured in 0.1 M KOH (aq) at room temperature. (*Specific current* is defined here as the magnitude of the current normalized to the catalyst mass. This is by analogy to the terms *specific energy* and *specific power* widely used in battery and fuel cell specifications.) Nafion perfluorosulfonic acid (PFSA) ionomer was used as a catalyst binder due to its wide use in thin-film RDE studies of PEM electrolyzer and fuel cell catalysts.^20^ Specific current increases across these catalysts in the order Ni/C < Ni–Mo/C < Pt/C < Pt–Ru/C, which is expected based on prior studies of these catalysts in alkaline aqueous electrolytes.^12,21^

Figure 1a further includes a simulated curve annotated “concentration overpotential” depicting the expected specific current vs potential relationship under conditions in which the rate of the HER is limited only by the transport of reactant/product species in the vicinity of the electrode surface.^8,22^ Notably, even the most active catalyst we studied, Pt–Ru/C, deviates from this curve at specific currents exceeding a few mA/mg. The data for Pt/C and Pt–Ru/C also take on a roughly linear trajectory in specific current vs overpotential for cathodic specific currents exceeding 20 mA/mg, which could be attributable to mass-transfer limitations or series resistance that results from the nucleation of microbubbles in the aqueous electrolyte at steady-state.^23^ We therefore carried out additional measurements while varying the catalyst mass loading to better understand the impact of catalyst layer thickness on apparent HER activity.

Figure 1b compiles data tracing out the relationship between catalyst mass loading and cathodic specific current at 100 mV overpotential. The specific current increases with reduced loading in all cases, and similar observations have been made by others when measuring HER activity of precious metals in acid and base.^8,21^ This behavior is attributable to the development of a concentration gradient in reactant/product species within the thickness of the catalyst film that translates to reduced catalytic turnover for active sites that are “buried” in the thin film. Thus, for sufficiently thin films, the specific current would be expected to converge on a single value that reflects an intrinsic kinetic limitation. Indeed, Ni–Mo/C and the two Pt-based catalysts exhibited evidence for a plateau in specific current as catalyst loading was reduced below 0.1 mg/cm^2^. No such plateau was observed for Ni, which we rationalize on the basis that Ni exhibits sufficiently low HER activity that the modest background current from the glassy carbon substrate (which does not diminish with catalyst loading) begins to dominate as catalyst loading decreases.

The dashed lines in Figure 1b reflect a fit of the empirical data to a mathematical relationship derived from the well-known concept of effectiveness factor in heterogeneous catalysis, with an additional term to account for substrate current (see Supporting Information for full details). From the fits we derived estimates for the kinetically limited specific current at 100 mV overpotential, *J*_*m*|η=0.1*V*_ Combining these data with TEM measurements of catalyst particle size (see Supporting Information) enables estimates to be made of the turnover frequency, *TOF*_η=0.1*V*_, based on assumptions that particles are perfectly spherical, all surface sites are equally active, and the density of surface sites is well-approximated by the bulk density of the respective metals (pure phases for Pt and Ni and fcc solid solutions for Ni–Mo and Pt–Ru). The resulting TOF estimates are compiled in Table 1.

**Table 1:** HER activity metrics for Ni- and Pt-based catalysts under pure kinetically controlled regime

**Catalyst**	**SSA** (*m*^2^/*g*)	**J**_*m*|η=0.1*V*_ (*mA*/*mg*_*cat*_)	**TOF**_η=0.1*V*_ (*s*^–1^)
40 wt % Ni/C	24	1.4	0.01
50 wt % Ni–Mo/C	140	150	0.60
60 wt % Pt/C	50	1020	8.4
60 wt % Pt–Ru/C	110	2050	7.9

While the measurements above facilitate comparisons of the “intrinsic” HER activity for each of these catalysts, the assumptions underlying the TOF values reported in Table 1 mean they should be regarded as rather imprecise estimates. Indeed, our results imply the intrinsic HER activity (TOF) of Pt and Pt–Ru are nearly identical, which disagrees with numerous prior reports demonstrating that the addition of Ru increases the activity of Pt toward alkaline hydrogen evolution/oxidation by at least 5 times.^24−28^ However, results compiled by Alia and Pivovar suggest that carbon-supported Pt and Pt–Ru nanoparticles exhibit much smaller differences in exchange current density.^21^ Our estimates were also made at 100 mV overpotential, which may be sufficiently large to introduce localized transport limitations that are not accounted for by the model in Figure 1b. Despite these uncertainties, the observation that Ni–Mo composites are only ∼ 10 times less active than Pt–Ru suggests the gap in practical performance (i.e., geometric current density at given polarization) could be narrowed further by increasing the loading of the (much less costly) nonprecious catalyst.

Motivated by promising activity data, we took further steps to develop catalyst ink formulations to compare Ni–Mo/C and Pt–Ru/C in lab-scale AEMWE devices. This prompted a transition from PFSA to anion-exchange ionomers as catalyst binders to better facilitate hydroxide transport across the AEM. Polyaryl piperidinium (PAP, Versogen PiperION) ionomers and membranes were chosen for the high hyroxide ion conductivity and durability they have shown in AEMWE assemblies to date.^29^

Figure 2 illustrates an unexpected result, where we observed a 10-fold decrease in cathodic specific current for Ni–Mo/C catalysts when using commercial PAP ionomers compared to Nafion PFSA in thin-film RDE measurements. Analogous measurements were made using carbon-supported Pt, Ni, Cu, and Ni–Cu catalysts and several other ionomer chemistries while varying the catalyst/ionomer ratios (full details in the Supporting Information). The poisoning effect was found to be unique and exclusive to the combination of Ni–Mo/C and the PAP ionomer.

**Figure 2:**
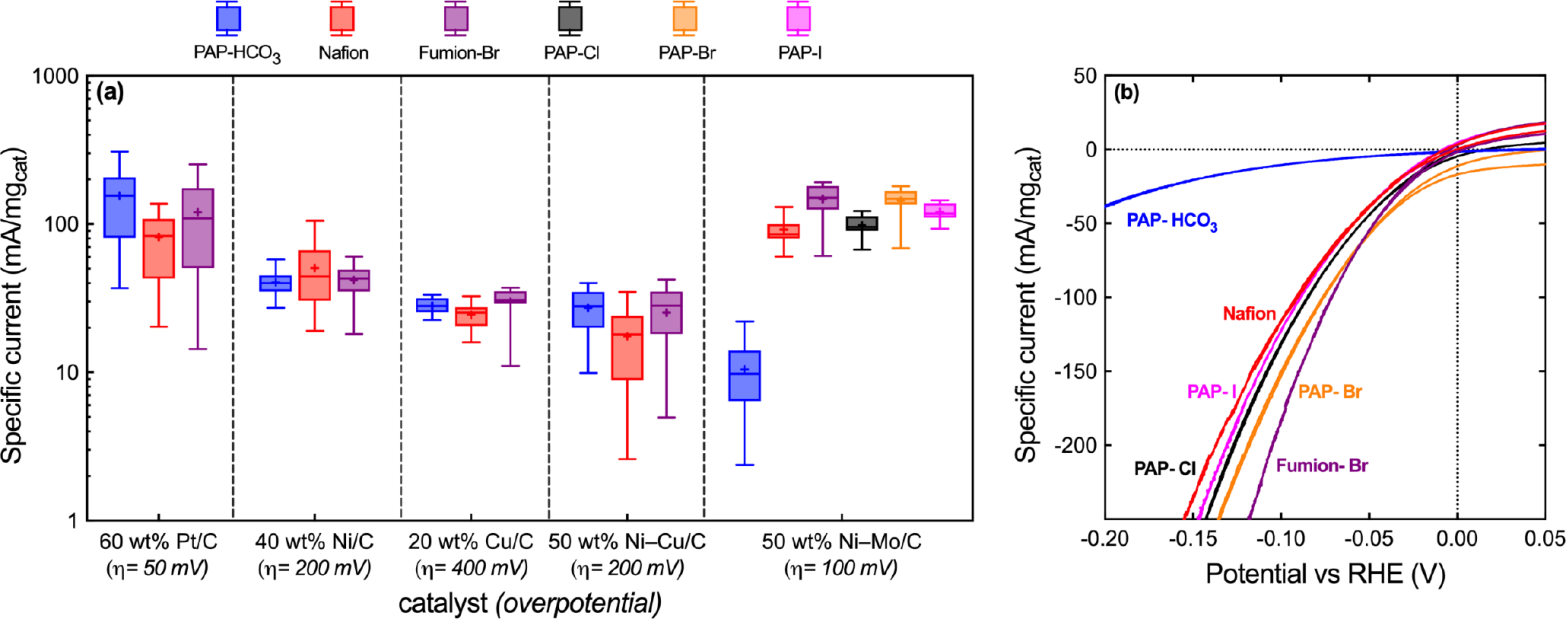
Compiled data summarizing studies of catalyst-ionomer interactions using five different alkaline HER catalysts. (a) Cathodic specific current densities for the noted catalyst-ionomer compositions and the noted overpotentials collected using thin-film RDE methods at 1600 rpm in 0.1 M KOH. These data are presented as box-and-whisker plots depicting the data mean (line), median (plus sign), intraquartile range (boxed regions), and maximum/minimum values (whiskers) from a minimum of 9 measurements under each condition. (b) Representative polarization curves for 50 wt % Ni–Mo/C at metal mass loading of 0.1 mg/cm^2^ and catalyst-to-ionomer ratio of 5:1. Labels for the hydroxide exchange ionomers (Fumion and PAP) are annotated with the identity of the charge-compensating counteranion in the catalyst ink.

Notably, the cathodic specific current of Ni–Mo/C was observed to be similar and comparatively high when using Nafion PFSA and Fumion FAA-3 hydroxide exchange ionomer, where the latter shares in common with the PAP ionomer the use of quaternary ammonium groups as the fixed cation and an aromatic polymer backbone. However, a key difference is that the Fumion ionomer solution was stabilized with bromide counter-anions, whereas the PAP ionomer used bicarbonate anions. Figure 2b therefore includes additional data we collected for Ni–Mo/C catalyst films using PAP ionomer binders with bicarbonate, chloride, bromide, and iodide counter-anions. Remarkably, the observed HER activity recovers for catalyst films generated with halide-stabilized PAP ionomers.

These results are unintuitive considering the well-known poisoning effect of halide ions on precious-metal based electrocatalysts.^30^ Work is ongoing to elucidate the molecular basis for reduced HER activity from Ni–Mo/C composites using the bicarbonate-stabilized PAP ionomer. Nonetheless, a key conclusion is that ionomer chemistry can have major impacts on catalytic activity, even for measurements made in liquid alkaline electrolytes where the ionomer is not solely responsible for ion transport. Moreover, the compatibility of an ionomer binder with a particular catalyst of interest clearly cannot be inferred based on its behavior with other catalysts.

With improved catalyst ink formulations in hand, we transitioned to measurements of full water electrolysis. Our focus was on AEMWE devices fed with aqueous alkaline electrolyte (0.1 M KOH), which avoids well-established challenges related to ion-transport in AEM ionomers fed with pure water and remains technologically tractable as a potential advance on liquid alkaline water electrolysis.^31,32^Figure 3 compiles polarization data directly comparing Ni–Mo/C and Pt–Ru/C cathodes in full AEMWE cells. The methods used to fabricate and test membrane-electrode assemblies are compiled in the Supporting Information. Ni–Mo/C cathodes were loaded with 1 mg/cm^2^ of total metal, whereas Pt–Ru/C was loaded at 0.15 or 0.3 mg/cm^2^. This difference reflects the ability to optimize independently around cost, activity, and transport behavior (i.e., active layer thickness) for precious and nonprecious electrolyzer catalysts. Preparation and testing procedures were otherwise identical in each case, as was the composition and loading of Ir oxide anodes. This allowed us to isolate differences in device performance that were specific to the composition and loading of the cathode catalyst.

**Figure 3:**
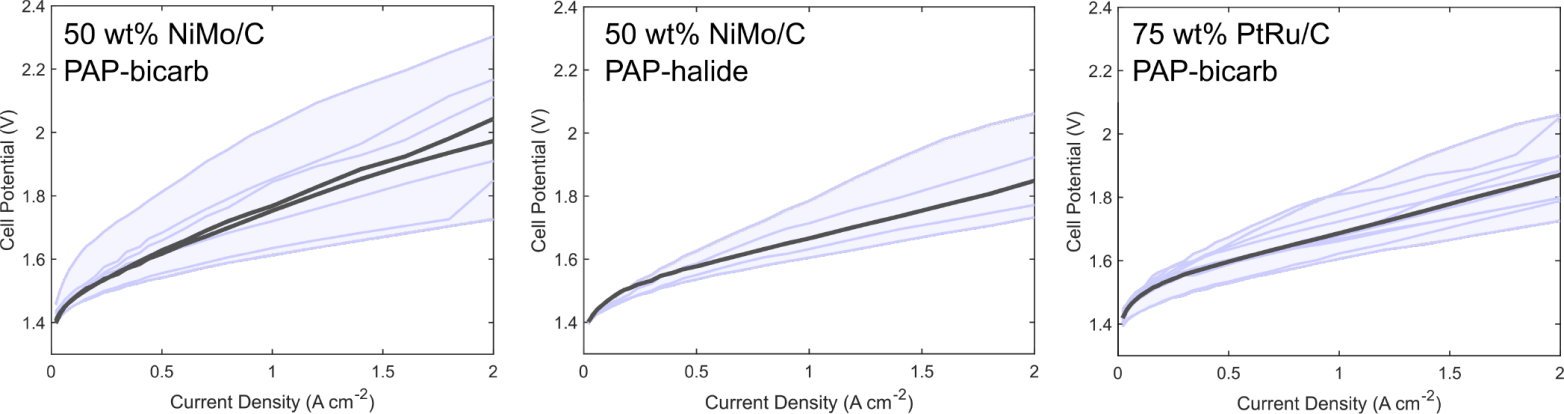
Compiled polarization curves for AEM electrolyzer assemblies using the noted cathode catalyst and binder, along with IrO_*x*_ anodes and PAP anion-exchange membranes, fed with 0.1 M KOH (aq). The median polarization curves (in terms of average cell potential over the full range from 0–2 A cm^–2^) are drawn in dark gray, with all other data sets depicted in blue. Note that the left panel depicts two median curves because we collected an even number of data sets.

A prevalent practice in the water electrolysis literature involves reporting single polarization curves under a given set of conditions without explicit discussion of replicates or error bounds.^33−37^ This is understandable in light of the time and resource intensity of such measurements, but it risks drawing conclusions from data that might ultimately prove to be outliers. Thus, Figure 3 depicts polarization curves for all 24 AEMWE assemblies that were used for this comparative study. Median curves are highlighted in gray and confidence intervals are demarcated in the shaded blue regions bounded by the best- and worst-performing devices we tested of each type. Statistical metrics like standard deviations or mean standard errors applied to these polarization curves would generally result in narrower confidence intervals, but they would be misapplied in this case because the datasets are not large enough to infer probability distributions.

Reporting results in this way enables several observations to be made. First, device performance varied considerably for all cathode catalysts studied—for example, cell potentials at 2 A/*cm*^2^ current density varied by >250 mV in each case. This is broadly consistent with the level of variability we routinely observe from AEMWE devices that are fabricated manually and incorporate novel materials (catalysts, membranes, polymer binders) for which optimized synthesis and processing methods have not yet been developed.

Second, AEMWE assemblies incorporating Ni–Mo/C cathodes derived from inks formulated with PAP ionomer in the bicarbonate form exhibited larger spread than those prepared with PAP ionomers in the halide form. Hence, onset potentials and “champion” device performance were nearly the same in each case, but cell polarization for the median PAP-bicarbonate MEAs exceeded that of PAP-halide MEAs by 50–150 mV at current densities above 500 mA/cm^2^. These results suggest the negative impact of the bicarbonate-based ionomer is related to inhibition of ion transport rather than catalyst deactivation, and the effect appears to be reduced significantly for cathodes in full AEMWE assemblies.

Finally, datasets collected for AEMWE cells using 1 mg *cm*^–2^ Ni–Mo/C and 0.15–0.3 mg *cm*^–2^ Pt–Ru/C cathodes are highly congruent. The median polarization curves differ from one another by no more than 25 mV from 0–2 A/cm^2^ current density, which is far smaller than the spread between the best and worst-performing MEAs in each case. Considering the only difference between these sample populations was the identity and loading of the cathode catalyst, these data strongly suggest that the cathode is not the underlying source of sample-to-sample variability. Indeed, one would expect this to be the case for Pt–Ru/C cathodes based on the very small kinetic polarization (<100 mV) expected for this precious metal catalyst. The best MEA performance we recorded for Ni–Mo/C and Pt–Ru/C also compares favorably with prior reports on electrolyzer assemblies based on PGM anode and cathode catalysts and fed with dilute alkali.^38−41^ Indeed, 19 of the 24 AEMWE assemblies (10 of 13 with Ni–Mo/C cathodes and 9 of 11 with Pt–Ru) included in this study exceed the 2026 performance target established by the U.S. Department of Energy for liquid alkaline electrolyzer cells.^42^

Note that the congruent performance of AEMWE assemblies Figure 3 does not imply the intrinsic activity (i.e., potential-dependent turnover frequency) of Ni–Mo/C and Pt–Ru/C is the same when the catalysts are incorporated into devices. To the contrary, Figure 1 cleary shows that Pt and Pt–Ru nanoparticles exhibit higher HER specific currents than Ni–Mo under modest HER polarization. A better interpretation of our findings is that neither Ni–Mo/C or Pt–Ru/C HER kinetics are the dominant contributors to overpotential losses (or sample-to-sample variability) in AEMWE cells. In fact, in both cases cathode polarization is clearly low compared to the total polarization, as evidenced by cell potentials of 1.4–1.5 V in the onset region around 10–100 mA cm^–2^, which is consistent with reported onset overpotentials for oxygen evolution at Ir oxide.^43^ Further reducing overpotential losses associated with ion transport and the oxygen evolution reaction could result in the ability to resolve differences in practical HER activity between these cathodes. Nonetheless, our results show PGM cathodes can be replaced with nonprecious alternatives in AEMWE assemblies while delivering initial performance that exceeds near-term performance targets. Further improvements are very likely achievable with additional efforts to tune Ni–Mo/C composition and microstructure to maximize the density of active sites.

Work continues in our lab to evaluate the durability of Ni–Mo/C under extended testing. To this end, we note the AEMWE measurements presented here were completed after a break-in procedure comprising a minimum of 12 h of continuous electrolysis at 100 mA/cm^2^. Moreover, prior measurements of unsupported Ni–Mo catalysts in liquid alkaline electrolyzers have demonstrated thousands of hours of continuous operation,^44^ implying degradation of full AEMWE devices incorporating Ni–Mo/C cathodes are likely be dominated by deleterious reactions at the anode.^32^ This possibility, in turn, motivates the continued development of analytical platforms that are capable of isolating half cells for performance and degradation measurements under technologically relevant conditions.^45,46^

In summary, the measurements of Ni/C, Ni–Mo/C, Pt/C, and Pt–Ru/C HER cathodes presented here offer useful insights into the relationships between intrinsic catalytic activity and practical performance. In agreement with prior reports, we found nanoparticulate Ni and Ni–Mo to be less active alkaline HER catalysts than Pt and Pt–Ru, but the difference between Ni–Mo and Pt–Ru is small enough that functional AEMWE assemblies constructed with either catalyst deliver nearly indistinguishable initial polarization performance. We further found that Ni–Mo/C suffers from a unique poisoning effect when combined with polyaryl piperidinium ionomers bearing bicarbonate anions, which illustrates that analytical protocols developed to estimate the instrinsic activity of one catalyst (e.g., Pt) cannot be unambiguously applied to all others. Hence, we conclude by offering an (admittedly immodest) recommendation to the research community to consider adopting Ni–Mo/C as a benchmark and platform upon which to develop ionomer binders, ink formulations, and device fabrication procedures for nonprecious cathodes in alkaline electrolyzers.

## Supporting Information Available

Experimental procedures, ancillary characterization data, and complete polarizaton data sets for catalyst-ionomer interaction measurements (PDF)
