# Supplementary material for: Comparing Intrinsic Catalytic Activity and Practical Performance of Ni- and Pt-Based Alkaline Anion Exchange Membrane Water Electrolyzer Cathodes
Source: ACS Energy Lett. 2025 Mar 18;10(4):1779–85. doi: 10.1021/acsenergylett.5c00439 (PMC11998071; doi:10.1021/acsenergylett.5c00439)
Supplement: Supplementary file 1 — nz5c00439_si_001.pdf [file nz5c00439_si_001.pdf]

# Supporting Information: Comparing Intrinsic Catalytic Activity and Practical Performance of Ni- and Pt-based Alkaline Anion Exchange Membrane Water Electrolyzer Cathodes

Advay Shirwalkar,[†] Manjodh Kaur,[†] Sichen Zhong,[‡] Max Pupucevski,[‡] Keda Hu,[¶] Yushan Yan,[¶] Judith Lattimer,[‡] and James McKone[*,†,§]

†*Department of Chemical and Petroleum Engineering, University of Pittsburgh, Pittsburgh, PA 15261, USA*

‡*Giner Labs, Newton, MA 02466, USA*

¶*Versogen, Newark, DE 19711, USA*

§*Department of Chemistry, University of Pittsburgh, Pittsburgh, PA 15261, USA*

E-mail: jmckone@pitt.edu

## Experimental Methods

### Material and Reagents

The reagents used in this study were obtained from commercial sources and employed without further purification unless specified otherwise. Nickel(II) nitrate hexahydrate (98%), platinum-ruthenium supported on carbon black (60 wt%, Pt:Ru = 2:1) and diethylene gly-

col (99%) were sourced from Alfa Aesar. Ammonium molybdate tetrahydrate (ACS reagent grade) was provided by ACROS Organics. Fisher Scientific supplied ammonium hydroxide (28-30 w/w%, ACS reagent grade) and isopropyl alcohol (99.5%), while ethanol (99.5%) was obtained from Decon Laboratories. Carbon-supported nickel nanoparticles (40 wt%), carbon-supported copper nanoparticles (20 wt%), 10 wt% Fumion (FAA-3) solution in NMP and carbon black (Vulcan XC-72) were purchased from Fuel Cell Store. Sulfuric and nitric acids (ACS Plus grade) were supplied by Fisher Chemicals. 5 wt% poly aryl piperidinium, PAP ionomers stabilized with carbonate and halide anions and dispersed in ethanol were supplied by collaborators from Versogen. Potassium hydroxide (ACS reagent grade) was acquired from Sigma Aldrich, and the Nafion ionomer solution (20 wt% dispersed in a water/alcohol mixture) was provided by Ion Power. Nickel foil (0.005 in thick, ≥99%) came from Alfa Aesar. Forming gas (5% $H_2$ and 95% $N_2$) and ultrapure $H_2$ (99.999%) were obtained from Matheson. Deionized water, with a resistivity of ≥18.2 MΩ·cm and total organic content of ≤3 ppb, was purified using a Millipore Advantage A10 system.

### Synthesis of Ni–Mo/C nanocomposite

The synthesis procedure was adapted from a previous study[S1] and modified by hydrothermal pre-oxidation of the carbon support to improve the dispersion of metal nanoparticles. In a typical synthesis, 1 g of carbon black (Vulcan XC-72, Fuel Cell Earth) was dispersed in 20 mL of deionized water within a 100 mL beaker. The dispersion was briefly sonicated until a homogenous, slurry-like suspension was achieved. Subsequently, 2 mL of 2 M aqueous nitric acid and 2 mL of 2 M aqueous sulfuric acid were gradually added to the suspension, which was stirred continuously at room temperature and 600 rpm for 1 hour to prevent agglomeration of the carbon particles. The resulting mixture was transferred to a Teflon-lined, bomb-style autoclave reactor, which was filled to three-fourths of its total volume with the prepared suspension, and the remaining space was filled with deionized water. The autoclave was securely sealed and placed in a preheated oven (Isotemp, 1660 W, Fisher Scientific) at 120°C

for 18 hours. After thermal treatment, the autoclave was carefully cooled in a fume hood. The cooled suspension was then collected by centrifugation (Sorvall ST8, Fisher Scientific) at 3000 rpm for 5 minutes and repeatedly washed with deionized water until the pH of the supernatant reached approximately 5, as measured with pH paper. The final product was dried in an oven (Isotemp, 1660 W, Fisher Scientific) at 60°C.

In a typical Ni–Mo/C synthesis aimed at achieving 50 wt% metal content, 22.5 mL of diethylene glycol (99%, Alfa Aesar) was introduced into a 100 mL beaker, followed by the addition of 0.1 g of dry oxidized carbon. The mixture was subsequently sonicated in an ultrasonic bath (Branson Ultrasonics Series M) to form a homogeneous slurry. In a separate beaker, a precursor solution was prepared by dissolving 0.75 g of nickel nitrate hexahydrate (98%, Alfa Aesar), 0.3 g of ammonium molybdate dihydrate (ACS reagent grade, ACROS Organics), 2.5 mL of deionized water (resistivity $\geq$18.2 M$\Omega$ cm, total organic content $\leq$3 ppb, purified using a Millipore Milli-Q Advantage A10), and 1 mL of ammonium hydroxide solution (28–30 w/w%, ACS reagent grade). This blue precursor solution was then added all at once to the carbon/glycol slurry and stirred for 1 minute at room temperature.

The resulting mixture was transferred onto a preheated hotplate set to 400°C, with the reaction temperature monitored using a thermometer. A mixed-phase Ni–Mo oxide precipitated as the mixture was heated to 110°C, after which the beaker was removed from the hotplate and allowed to cool for several seconds. The warm solution was centrifuged at 3000 rpm for 8 minutes, then washed and resuspended 2–3 times in water and acetone. Subsequently, the carbon-supported Ni–Mo oxide catalyst precursor was rinsed with methanol (99.5%, Fisher Scientific) and dried at 60°C. Finally, the catalyst precursor underwent reduction in a single-zone tube furnace (Lindberg Mini-Mite, Fisher Scientific) at 200°C for 30 minutes and 450°C for 1 hour under a forming gas atmosphere (5% $H_2$, 95% $N_2$, Matheson gas), yielding the final catalyst.

### Synthesis of Ni–Cu/C nanocomposite

Ni–Cu/C was synthesized by a similar method as with Ni–Mo/C. Briefly, a total of 210 mg of hydrothermally oxidized Vulcan carbon was dispersed in 10 ml of deionized water using a homogenizer to achieve a uniform dispersion, which was then subjected to continuous stirring. A separate solution was prepared by dissolving 421 mg of Ni(II) nitrate hexahydrate and 54 mg of Cu(II) nitrate hemi-pentahydrate in 5 ml of deionized water. This solution was then gradually added dropwise to the carbon dispersion under stirring. The resulting mixture was homogenized for 1 minute and subsequently dried in an oven at 105°C overnight. The obtained black solid was then thermally reduced under forming gas at 550°C for 1 hour, with the furnace programmed to reach the target temperature over a span of 2 hours.

## RDE test protocol

### Catalyst ink formation and electrode preparation

The rotating disk electrode (RDE) test protocol began by weighing 5 mg of 20 wt% Nafion dispersion into a scintillation vial. 1 mL of ethanol was added to the vial, and the solution was sonicated for 5 minutes to ensure thorough mixing of the ionomer and solvent. Next, 5 mg of the desired catalyst was measured and added to the prepared solution. To achieve proper dispersion of the catalyst, the mixture was further sonicated for an additional 15 minutes.

The ionomer content was adjusted as necessary to achieve the desired catalyst-to-ionomer ratio, with a default ratio of 5:1 (catalyst powder mass to ionomer mass) used unless stated otherwise. The resulting ink was dropcast onto a 5 mm diameter glassy carbon electrode with a Teflon shroud (Pine Research) using a 10 $\mu$l repeating pipettor (Thermo Scientific F1 ClipTip) and allowed to dry under an infrared lamp.

### Investigation of Ionomer Effects on Catalyst Performance

The rotating disk electrode (RDE) tests were performed utilizing three distinct ionomers: Nafion, poly(aryl piperidinium) (PAP-$HCO_3$), and Fumion (FAA-3). Five catalyst-to-ionomer ratios were systematically explored: 20:1, 10:1, 5:1, 2.5:1, and 1:1. The catalysts employed in these investigations included 60 wt% Pt/C (obtained from Alfa Aesar); 40 wt% Ni/C and 20 wt% Cu/C (obtained from Fuel cell Store); and 50 wt% Ni–Mo/C and 50 wt% Ni–Cu/C (synthesized in house using the methods described above). The experiments were conducted at a consistent rotation speed of 1600 rpm within a hydrogen-saturated 0.1 M aqueous KOH solution, with a scan rate of 10 mV/s. The metal mass loading on the electrode was consistently maintained at 0.1 $mg_{cat}$ $cm^{-2}$ unless specified otherwise. Full results for all ionomer loading studies are compiled in Figure S1.

## Experimental protocol for mass-loading studies

Randomized multistep chronoamperometry was performed to determine the current response at specific potential values for a library of catalysts. This technique recues the impact of non-faradaic currents. The ink preparation followed the same procedure as previously described, but used inks with higher solids content. Approximately 40 mg of catalyst powder was added to a pre-sonicated ethanol solution containing 160 mg of 5 wt% Nafion dispersion (obtained from Fuel cell Store). This mixture was sonicated for approx. 10 minutes. A serial dilution step was then performed to prepare inks of lower concentrations, with a dilution ratio of approximately 10:1. All these inks were continuously sonicated in an ice-cooled bath throughout the duration of experimentation to avoid particle settling and agglomeration and solvent evaporation. These inks were then sequentially used to dropcast a known volume onto the electrode and tested in a 0.1 M KOH solution on an rotating disk electrode (RDE) instrument at 1600 rpm under hydrogen purge.

Raw data collected from chronoamperometry measurements featured significant noise at high current values, attributable to the formation and detachment of hydrogen bubbles

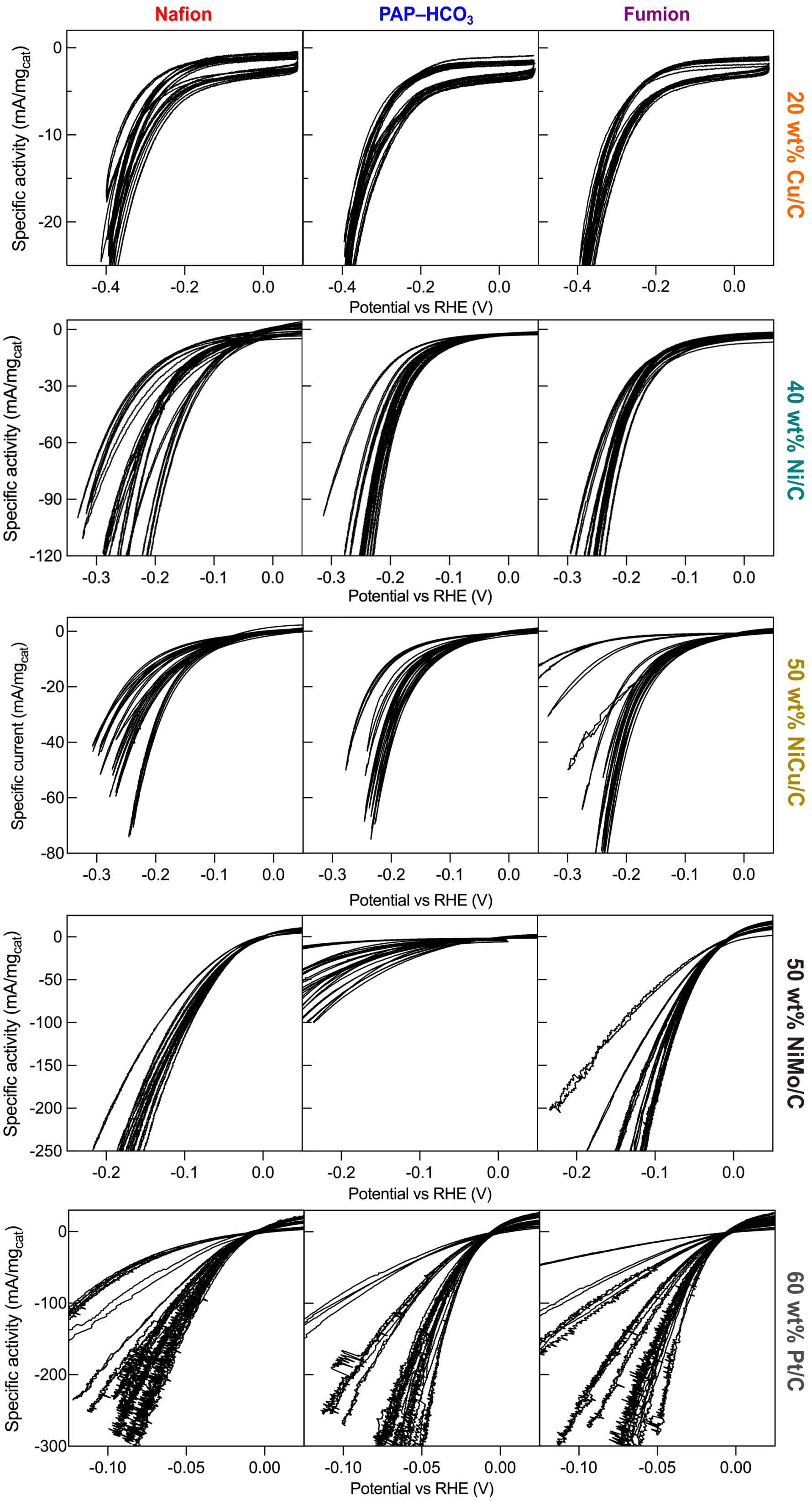


Figure S1: Polarization curves for the full library of catalysts and ionomers tested to generate the results in Figure 2 of the main text. All catalysts were tested at a loading of 0.1 $mg_{cat}/cm^2$.

at the electrode surface. Steady-state current values were extracted from the data using the procedure outlined below and schematized in Figure S2. First, data from the first 5 seconds at a given potential weree discarded to remove the effects of capacitive charging. The remaining data was then binned into a histogram reflecting the distribution of current values observed at the target overpotential. The most frequent value of the current was then selected from the histogram and used as the basis of the steady-state polarization analysis in maintext Figure 1a. These data were further corrected for electrolyte series resistance using empirically measured values from impedance spectroscopy, and values were linearly interpolated between individual datapoints as needed to extract quantitative activity metrics, e.g., at 100 mV overpotential. This approach presupposes that the observed catalytic current is randomly distributed about a "true" value, such that the most prevalent value in the histogram reflects the central tendency. In fact, this approach may underestimate the kinetically limited current, as the central tendency likely reflects a condition in which the catalyst-electrolyte interface is partially covered with gas bubbles.

These interpolated specific currents were plotted against the corresponding mass loading and fit to a three-parameter hyperbolic equation of the form:

$$J_m = a\frac{\tanh(b \cdot m)}{b \cdot m} + \frac{c}{m} \tag{1}$$

where $J_m$ is specific current in $mA/mg_{cat}$ at mass loading $m$ in $mg_{cat}/cm^2$ and $a$,$b$ and $c$ are the fitted parameters. The parameters were obtained through non-linear regression with the sum of the normalized error as the objective function, which allows for scaling all the errors. The first term in Equation 1 was derived from the well-developed concept of effectiveness factors in heterogeneous catalysis, which accounts for the impact of mass-transfer through a porous catalyst layer.[S2] It predicts an increase in $J_m$ to a maximum value (which is defined by the parameter $a$ in Eq. 1) as mass loading decreases—this maximum corresponds to the specific current for an infinitely thin catalyst layer with negligible transport limitations.

Hence, the fitted asymptotic values of $a$ were reported as "intrinsic" specific current and used to estimate TOF values for maintext Table 1. The second term $\frac{c}{m}$ in Eq. 1 accounts for the presence of a constant background current $c$, which must be much smaller than $a$ to facilitate an accurate estimate of the kinetically limited specific current.

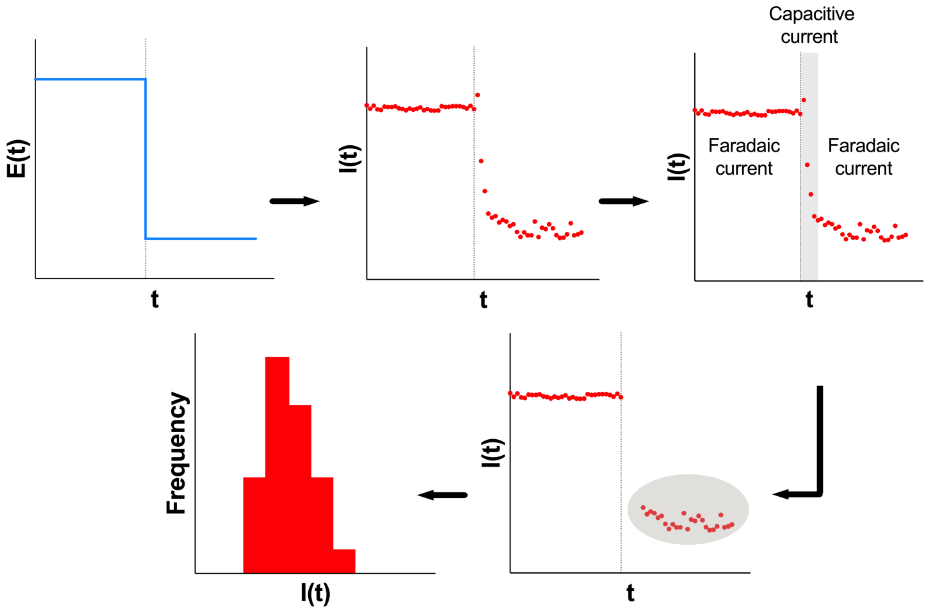


Figure S2: Graphical illustration of the method by which current values were determined from multistep chronoamperometry measurements: data from the first 5 seconds of polarization after each potential step were discarded; the remaining data were binned into a histogram and the final values extracted as the most frequent current observed in the histogram.

Representative polarization data normalized to electrode surface area are depicted in Figure S3 and S4 as linear and semilog (Tafel) plots. Exchange current densities ($j_0$) were estimated by performing a linear regression on the log(current density) versus potential plot (Tafel line) using all data collected at potentials below -0.1 V vs RHE. The resulting line was extended to the y-axis to obtain the $j_0$ value.

The concentration overpotential curve was modeled using the following equation:

$$\eta_{conc} = \frac{1}{nf}\left[ln\left(\frac{j_{a,lim}}{-j_{c,lim}}\right) - ln\left(\frac{j_{a,lim}-j}{j-j_{c,lim}}\right)\right] \tag{2}$$

where the cathodic and anodic limiting currents were calculated using the following formula:

$$j_{lim} = 0.62nFC_{bulk}D^{2/3}\omega^{1/2}\nu^{-1/6} \tag{3}$$

here $C_{bulk}$ is the bulk concentration of water for cathodic reaction (55.56 mol/L), while dissolved hydrogen for anodic reaction (0.0008 mol/L) in 0.1M KOH solution under ambient conditions; $D$ is the diffusion coefficient of water for cathodic portion and $H_2$ for anodic portion; $\omega$ is the rotation rate; and $\nu$ denotes the kinematic viscosity of water.

## Electrolyzer measurements

We conducted measurements of current density versus potential for full water electrolysis utilizing a single-cell anion-exchange membrane (AEM) electrolyzer. The experimental protocol was adapted from our previous publication.[S1] An 80 $\mu$m thick PiperION A membrane (Versogen) was employed along with the anion-exchange ionomer PiperION A (5 wt% in ethanol; Versogen) as the catalyst binder. The cathode catalyst was either a 50 wt% Ni–Mo/C loaded at 1.0 $mg_{cat}$ $cm^{-2}$ or a 75 wt% Pt–Ru/C (50 wt% Pt and 25 wt% Ru) loaded at 0.15 or 0.3 $mg_{cat}$ $cm^{-2}$, while the anode comprised unsupported nanoparticulate $IrO_2$ (2–3 $mg_{cat}$ $cm^{-2}$).

Catalyst films were prepared by manually air-spraying a suspension of catalyst ink consisting of 20 vol% water and 80 vol% isopropanol onto the anion-exchange membranes. The membrane electrode assemblies (MEAs) were pre-conditioned by soaking them in 3 M KOH for 1 hour prior to their assembly into a 5 $cm^2$ electrolyzer, utilizing H23C6 carbon paper (Freudenberg) as the cathode gas-diffusion layer (GDL) and platinized titanium sinter as the anode GDL. Further conditioning was carried out by flowing ultrapure water at a rate of 0.35 mL $min^{-1}$ through the anode while heating the cell to 80 °C and applying a current of 0.1 A $cm^{-2}$. This flow rate, which was maintained throughout the experiment, was empirically determined to support current densities exceeding 2 A $cm^{-2}$ without transport limitations. Conditioning continued until a steady-state voltage was achieved, which took approximately

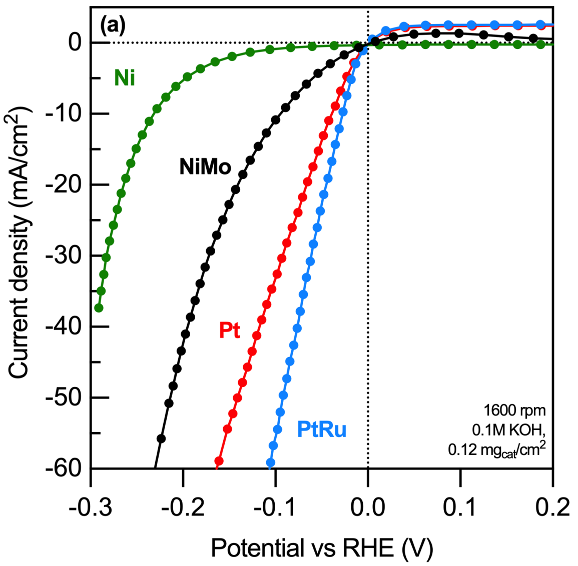


Figure S3: (a)Representative polarization data obtained from multi-step chronoamperometric runs at mass loadings of 0.12 $mg_{cat}/cm^2$ in 0.1M KOH solution at 1600 rpm.

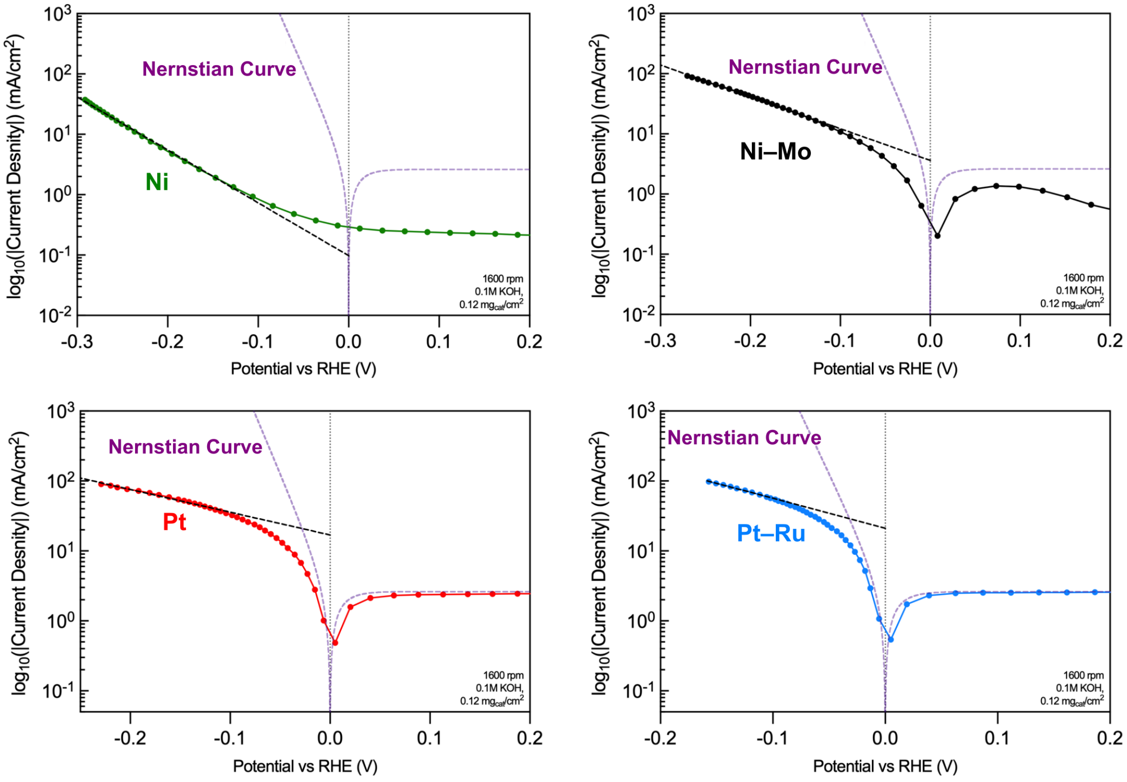


Figure S4: Same datasets as in Figure S3 depicted on semilog axes.

12–18 hours.

Following conditioning, polarization curves were recorded. Chronopotentiometric polarization curves were collected over a current range from 0.02 A $cm^{-2}$ to 2.0 A $cm^{-2}$ at 80 °C under anode feed conditions with a 0.1 M KOH electrolyte. Polarization curves were recorded at fixed currents for durations of 1–2 minutes to obtain stable cell voltages. Between collections of polarization curves, the cell was maintained at 0.1 A $cm^{-2}$. Figure 3 in the main text presents the polarization curves for each MEA tested under the specified composition and configuration. In instances where multiple polarization curves were collected for a given MEA, the reported data correspond to the curve demonstrating the highest performance, under the assumption that this reflects a fully broken-in device.

# Analytical Methods

## X-Ray diffraction analysis

X-ray diffraction (XRD) analysis was employed to confirm catalyst identity and to provide an initial estimate of nanoparticle size. XRD measurements were conducted using a Bruker D8 system with Cu K$\alpha$ radiation ($\lambda = 1.5406$ Å). For sample preparation, powdered samples were thoroughly pulverized to ensure uniformity, and then loaded onto a stainless steel stage via a back-loading method. All samples were analyzed under identical conditions, with a scan speed of 1.3 sec/scan and a step size of 0.05° per scan. Representative XRD data are shown in Figure S4.

## TEM and Particle size analysis

Transmission electron microscopy (TEM) was used to obtain a more precise estimate of particle size, which was then used for surface area and turnover frequency (TOF) calculations. Figures S5–S8 compilee representative TEM images along with particle size distributions obtained from manual measurements of particle diameters based on the TEM data.

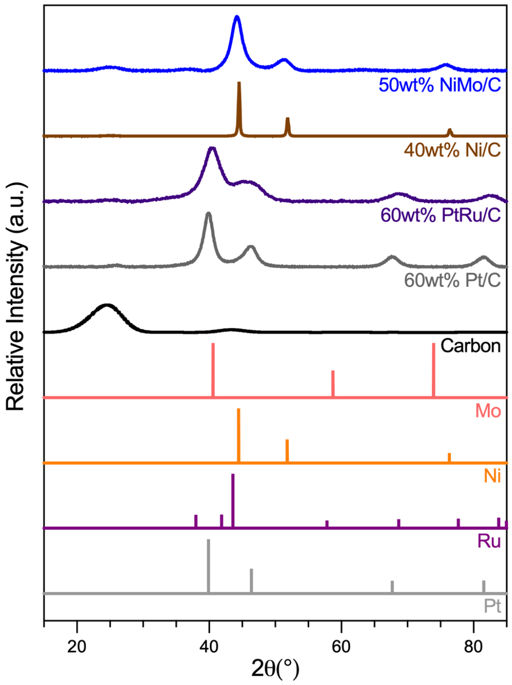


Figure S5: Representative X-ray diffraction data for the noted carbon-supported catalyst composites, with standard patterns obtained from ICDD.

Table S1 provides a comparison of particle size estimates derived from Scherrer analysis and TEM imaging. It is important to note that the TEM-derived results were employed to estimate specific surface areas for TOF calculations. The discrepancy between the TEM and XRD measurements of particle and crystallite size for Ni/C (it is not possible for a single crystallite to be larger than a single particle) may stem from the broad particle size distribution observed, as the Scherrer method yields a volume-weighted average, which typically produces a larger estimate compared to a particle-count-based distribution.

Table S1: Tabulated matrix of crystallite and particles size obtained through XRD and TEM respectively for a library of catalytic materials

| Catalyst material | Avg. crystallite size (XRD), *nm* | Avg. particle size (TEM), *nm* |
|---|---|---|
| 50 wt% NiMo/C | 4.65 | 7.69 |
| 40 wt% Ni/VC | 28.29 | 17.63 |
| 60 wt% Pt/C | 5.24 | 6.05 |
| 60 wt% PtRu/C | 3.25 | 3.65 |

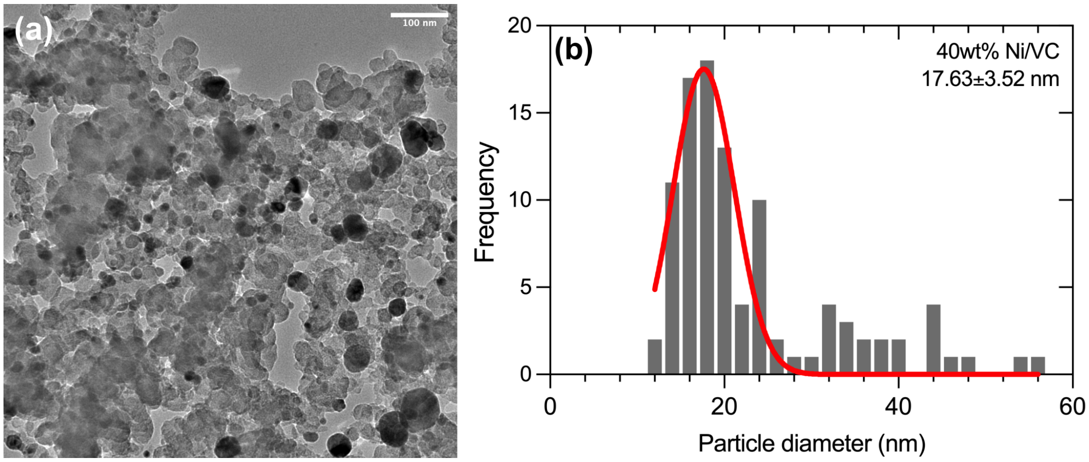


Figure S6: (a) Representative TEM image of 40 wt% Ni/C. (b) Particle size distribution for 100 particles measured from the adjacent TEM image.

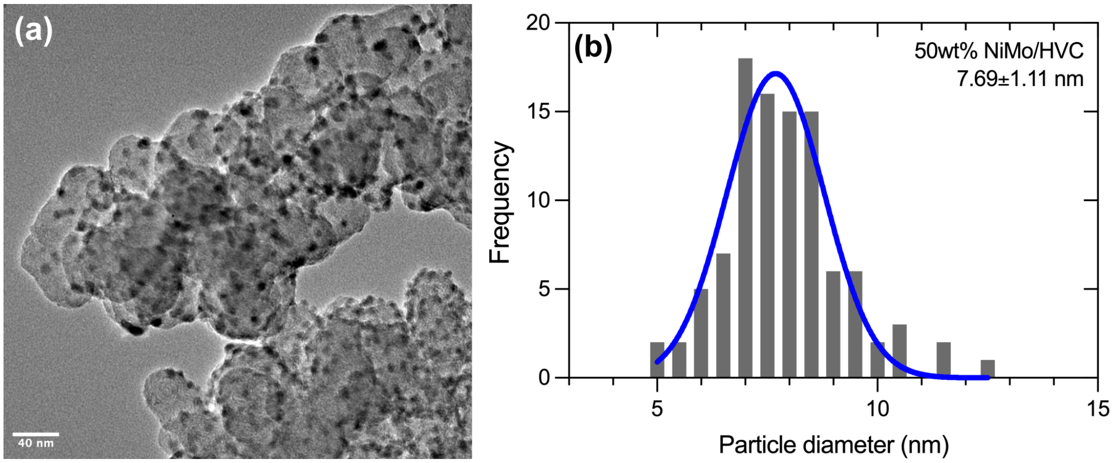


Figure S7: (a) Representative TEM image of 50 wt% Ni–Mo/C. (b) Particle size distribution for 100 particles measured from the adjacent TEM image.

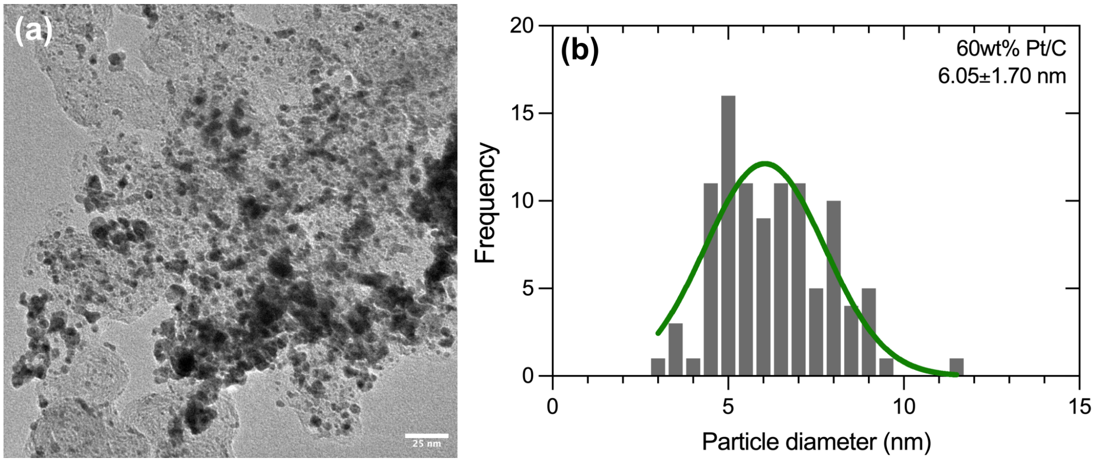


Figure S8: (a) Representative TEM image of 60 wt% Pt/C. (b) Particle size distribution for 100 particles measured from the adjacent TEM image.

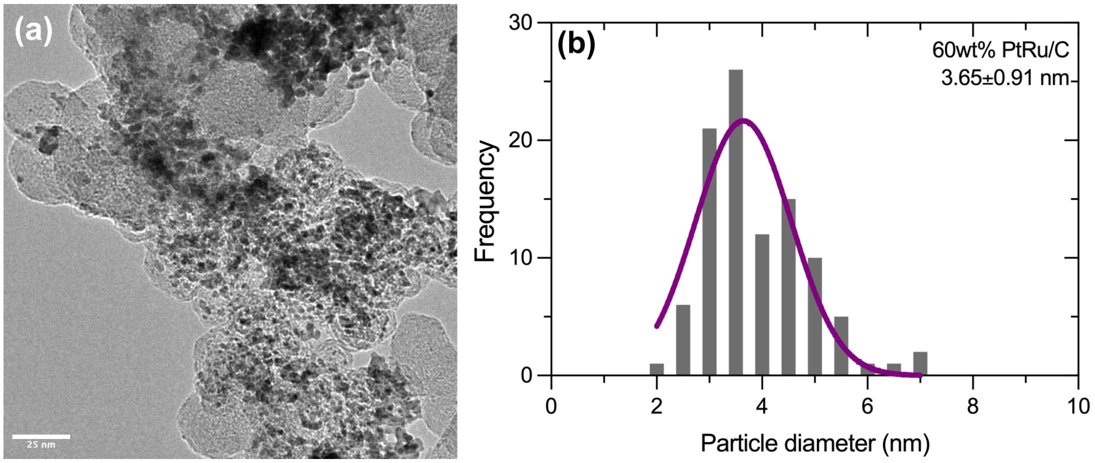


Figure S9: (a) Representative TEM image of 60 wt% PtRu/C. (b) The particle size distribution for 100 particles measured from the adjacent TEM image.

# References


(S1) Patil, R. B.; Kaur, M.; D. House, S.; Kavalsky, L.; Hu, K.; Zhong, S.; Krishnamurthy, D.; Viswanathan, V.; Yang, J.; Yan, Y.; Lattimer, J.; R. McKone, J. Reversible alkaline hydrogen evolution and oxidation reactions using Ni–Mo catalysts supported on carbon. *Energy Advances* **2023**, *2*, 1500–1511.

(S2) Levenspiel, Octave, *Chemical Reaction Engineering*, 3rd ed.; John Wiley & Sons, Inc, 1999.
